# Intravenous neutralization of vascular endothelial growth factor reduces vascular function/permeability of the ovary and prevents development of OHSS-like symptoms in rhesus monkeys

**DOI:** 10.1186/s13048-017-0340-5

**Published:** 2017-07-06

**Authors:** C. V. Bishop, D. M. Lee, O. D. Slayden, X. Li

**Affiliations:** 1 Division of Reproductive & Developmental Sciences, Oregon National Primate Research Center, Beaverton, OR 97006 USA; 2 Obstetrics & Gynecology, Oregon Health & Science University, Portland, OR 97239 USA; 3 Advanced Imaging Research Center, Oregon Health & Science University, Portland, OR 97239 USA

**Keywords:** VEGFA, Chorionic Gonadotropin, Controlled ovarian stimulation, Ovarian vascular function, Uterine vascular function

## Abstract

**Background:**

Ovarian hyperstimulation syndrome (OHSS) is a disorder associated with elevated serum VEGFA following chorionic gonadotropin (hCG) exposure in controlled ovarian stimulation (COS) cycles in women. In this study, we tested the effect of intravenous VEGFA neutralization on OHSS-like symptoms and vascular function in rhesus macaques during COS cycles.

**Methods:**

Monkeys (*n* = 8) were treated with 3 COS protocols and assigned randomly to groups as follows: 1) COS alone (**Control,** *n* = 5); 2) COS + VEGF mAb Avastin 19 ± 5 h before hCG (**Avastin pre-hCG**; *n* = 6); 3) COS + Avastin 3–4 days post-hCG (**Avastin post-hCG**; *n* = 4); 4) COS + Simulated Early Pregnancy (**SEP** *n* = 3); or 5) COS + SEP + Avastin (**SEP + Avastin** *n* = 3). Follicles were aspirated 36 h post-hCG, fluid was collected from one follicle for analysis of steroid and vascular hormone content. Remaining follicles were aspirated, and luteinized granulosa cells (LGCs) cultured for 24 h. Ovarian/uterine vascular flow (VF) and blood volume (BV) were analyzed by contrast enhanced ultrasound (CEUS) before hCG bolus and 6–8 days post-hCG bolus/time of peak SEP response. Ovarian permeability to albumin was analyzed by Dynamic Contrast Enhanced-MRI (DCE-MRI) post-hCG.

**Results:**

Abdominal fluid was present in 4/5 Control, 2/6 Avastin pre-hCG, and 3/4 Avastin post-hCG females. Neutralization of VEGFA before hCG reduced ovarian VF, BV, and permeability to albumin (*P* < 0.05), while only ovarian VF and permeability were reduced in Avastin-post hCG group (*P* < 0.05). There was no effect of Avastin on ovarian vascular function during COS + SEP. VEGF levels in follicular fluid were reduced 78-fold by Avastin pre-hCG, and LGCs exposed to Avastin in vivo also released 4-fold less VEGF into culture media (*P* < 0.05). Culture medium of LGCs exposed to VEGFA neutralization in vivo had lower levels of P4 and ANGPT1, and an increased ratio of ANGPT2/1 (*P* < 0.05). Uterine VF was reduced by SEP + Avastin in the basalis/junctional zone (*P* < 0.05).

**Conclusions:**

Avastin treatment before hCG prevents the development of symptoms associated with ovarian hyperstimulation syndrome. In vitro data suggest neutralization of VEGFA alters expression of other vascular factors typically induced by hCG in the luteinizing follicle. Neutralization of VEGFA action alters the vascular function of the basalis zone of the uterus during simulated early pregnancy, indicating a potential effect on embryo implantation.

**Electronic supplementary material:**

The online version of this article (doi:10.1186/s13048-017-0340-5) contains supplementary material, which is available to authorized users.

## Background

Controlled ovarian stimulation (COS) protocols to obtain multiple oocytes are often used on women seeking treatment for infertility [1], and many administer chorionic gonadotropin (CG or hCG) to induce oocyte maturation. This hormone is biosimilar to luteinizing hormone (LH), both are heterodimeric glycoproteins that bind the same receptor (LHCGR): they share a common α subunit and only differ amino acid composition of the β subunits [2]. The larger β subunit of hCG renders the molecule more stable and results in a longer half-life in circulation compared to LH [2], making it a more practical hormone to use in COS protocols. While COS protocols allow for retrieval of multiple mature (MII-stage) oocytes increasing the odds of a successful pregnancy, these protocols also result in the formation of multi-luteal ovaries in these women. Induction of multiple antral follicles to luteinize leads to abnormally high production of angiogenic factors by the ovaries [3]. Local ovarian production of angiogenic factors induced by the mid-cycle surge of LH [4], specifically vascular endothelial growth factor A (VEGFA) and angiopoietins (ANGPTs), are required for formation of the vascular bed of the corpus luteum (CL): a network of small fenestrated (leaky) capillaries [5, 6] with high vascular flow [7]. However, the mechanisms involved in regulation of VEGFA/ANGPTs expression by LH or hCG remains to be definitively elucidated in primates. Overproduction of angiogenic factors is hypothesized to alter both the local and systemic vasculature and contributing to ovarian hyperstimulation syndrome (OHSS), a pathologic condition associated with hCG use in COS [8].

OHSS is a vascular disorder with clinical symptoms related to an overall increase in systemic vascular permeability [3]. In cases of moderate OHSS, fluid and serum proteins (albumin) are lost from the vasculature, leading to a buildup of ascites fluid in extravascular spaces/abdomen. If left untreated, moderate OHSS can progress to pleural effusion, thrombosis, and hypovolemic shock reported in cases of severe/critical OHSS [9]. OHSS is associated with hCG exposure during COS, an infertility diagnosis of polycystic ovarian syndrome (PCOS), high serum estrogen on the day of hCG administration, large numbers of follicles stimulated, and high numbers of oocytes retrieved [10]. OHSS can develop either in direct response to CG trigger, (Early Onset OHSS) or later in early pregnancy in response to endogenous CG, (Late Onset OHSS).

Recent clinical research has focused on modification of stimulation protocols to avoid OHSS [11] since many vascular processes contributing to the development of OHSS can affect embryonic implantation and placental development. Use of these modified stimulation protocols (gonadotropin releasing hormone (GnRH) antagonists, GnRH agonist trigger, cryopreserving embryos, etc.) will reduce risk, but do not eliminate OHSS cases in women undergoing gonadotropin stimulation [12] or in rare cases of spontaneous OHSS during pregnancy [13].

Rhesus monkeys provide an excellent nonhuman primate model to assess multi-follicular development [14]. In women with OHSS, high levels of VEGFA are postulated to be the main contributing factor to the development of the disorder [15–17]. Likewise, blockage of VEGFA actions within the ovary during ovulation in normal menstrual cycles of macaques impairs development and function of the luteal vasculature [6]. However, only associative studies have linked VEGFA actions with the development of primate ovarian hyperpermeability and OHSS symptoms [15, 16, 18]. Further research intro mechanisms controlling formation and function of the vascular bed of the CL in primates is critical to developing therapies to treat OHSS and clarify mechanisms contributing to normal luteal function.

These studies were designed to test the hypothesis that VEGFA is the main factor contributing to primate ovarian hyperpermeability and abdominal ascites following COS cycles resulting from either early or late exposure to hCG. Female rhesus monkeys undergoing COS were adminstered Avastin® (bevacizumab, Genentech, South San Francisco, CA, USA), a humanized monoclonal antibody targeting VEGFA used in conjunction with chemotherapy to prevent ascites associated with several cancers [19], by intravenous injection. This treatment was designed to block VEGFA actions throughout the entire body as well as the reproductive tract. The goal of this study was to determine if VEGFA neutralization with Avastin, either before or after hCG administration, would alter ovarian/uterine vascular function and prevent the development of symptoms associated with OHSS. Additionally, the role of hCG-stimulated VEGFA in the development of late onset OHSS symptoms was evaluated in macaques during simulated early pregnancy (SEP) following COS.

## Methods

Female reproductive-aged (8.3 ± 0.4 years old) rhesus macaques (*Macaca mulatta*; *n* = 8) were utilized in these studies. Animal care was provided by the Division of Comparative Medicine at the Oregon National Primate Research Center (ONPRC) [20]. All procedures were reviewed and approved by the ONPRC/Oregon Health and Science University (OHSU) Institutional Animal Care and Use Committee (IACUC) in accordance with the U.S. Public Health Service (PHS) Policy on Humane Care and Use of Laboratory Animals.

### Natural menstrual cycles

Estradiol (E2) and progesterone (P4) levels were monitored during the menstrual cycle beginning 6–8 days following onset of menstruation (cycle day 1). Once E2 levels rose above 150 pg/ml, indicating a large, preovulatory antral follicle was present, the animals (*n* = 8) underwent evaluation of ovarian structure by 3D/4D ultrasound and ovarian/uterine vascular flow and blood volume by contrast-enhanced ultrasound (CEUS) as previously described [7, 21]. In the mid-luteal phase, 6–8 days following mid-cycle E2/LH surge [7], luteal/uterine vascular function and ovarian permeability were evaluated again by CEUS, and at this time ovarian permeability to albumin was quantified by dynamic contrast-enhanced magnetic resonance imaging (DCE-MRI).

### Controlled ovarian stimulation (COS) cycles

Ovarian stimulation and follicle aspiration were performed using previously described methods [22] (*n* = 15 COS cycles; 2 cycles/female, 1 cycle was canceled). Briefly, near the onset of frank menses follicle stimulating hormone (FSH) was administered daily (60 IU/day) for 6 days. Then, LH (60 IU/day) was administered daily in conjunction with FSH (60 IU/day) until hCG (1000 IU) was administered to trigger oocyte maturation/follicle luteinization 8–9 days after start of COS. Ovarian structure and ovarian/uterine vascular function were analyzed by 3D/4D ultrasound and CEUS −0.7 ± 0.2 days prior to hCG bolus. The animals then oocyte retrieval/follicle aspiration 36 h following hCG bolus: contents of one randomly chosen follicle/female was aspirated by hand syringe technique and stored at −80 °C until hormone analyses, while the remaining follicles were aspirated and contents pooled by female for oocyte evaluation and cell culture as described below. Similar to natural menstrual cycles, luteal/uterine vascular function and ovarian permeability were analyzed by both CEUS and DCE-MRI 6–8 days following hCG administration.

### Simulated early pregnancy (SEP) following COS cycles

Seven to nine days following the COS protocols, the animals (*n* = 6) received increasing doses of hCG (15 to 360/750 IU) for 6–7 days to mimic an implanting embryo. This method prolonged luteal function similar to early pregnancy in rhesus females [23, 24]. Evaluation of ovarian/uterine vascular function (CEUS) and ovarian permeability to albumin (DCE-MRI) was performed the day before and at/near the time of peak P4 response to SEP.

### Avastin treatment during COS and SEP

Females received a single slow IV bolus of Avastin® (bevacizumab, Genentech, South San Francisco, CA, USA; 10 mg/kg) either 19 ± 5 h before hCG (*n* = 6 COS cycles) or 3.8 ± 0.3 days post-hCG bolus (*n* = 4 COS cycles). Homology between human and rhesus VEGFA is predicted to be 100% (NCBI Protein-Protein BLAST alignment, rhesus VEGFA and human VEGFA transcripts NCBI Gene ID: 574,209). This dose is reported to effectively reduce VEGF levels with minimal side effects in cynomologus macaques [25]. Animals also received an Avastin bolus the day before initiation of SEP following COS (−1; 10 mg/kg). Since serum concentrations of Avastin are reported to decline after 4 days [25], an additional IV bolus was administered 2.7 ± 0.3 days later in SEP cycles (10 mg/kg; SEP + Avastin, *n* = 3 cycles). Females showed no detrimental effects of Avastin treatment on serum hematocrit or other markers of well-being (rapid weight change, etc.; data not shown).

### Dynamic contrast-enhanced magnetic resonance imaging (DCE-MRI)

Immediately following ovarian/uterine evaluation by CEUS, animals underwent MRI scanning. Females were placed under isoflurane anesthesia to minimize movement during scanning. Abdominal planes were imaged with a Siemens Magnetom Trio 3 T whole-body MR instrument with Total imaging matrix (Tim) technology. The approximate location of the ovaries identified by ultrasound was landmarked with the scanner laser beam for subject positioning. Radio frequency (RF) transmitting utilized the built-in body coil, and RF receiving used a 15-element human knee RF coil. For structural imaging, a low-resolution sagittal scan (slice thickness 1 mm, space between slices 5 mm, echo time (TE) 20 ms, repetition time (TR) 500 ms, flip angle 90°) was used to determine the orientation of reproductive tract. A series of high-resolution transverse T2-weighted images/slices (in-plan resolution 0.5 mm, slice thickness 1 mm, space between slices 0.05 mm, TE 101 ms, TR 3610 ms, flip angle 90°) were obtained to visualize the ovaries, uterus, and presence of any ascites fluid. For contrast-enhanced MRI, dynamic scans (3 s intervals, slice thickness 2 mm, TE 1.94, TR 5.76, flip angle 12°) were obtained before, during, and after (wash out) an IV bolus of Ablavar (0.03 mmol/kg; gadofosveset trisodium, Lantheus Medical Imaging, N. Billerica MA, USA). Ablavar is bound to serum albumin [26], and thus transit of Ablavar through the tissue of interest can demonstrate permeability to albumin. To provide an MRI estimate of the blood pool, the same high-resolution T2-weighted scans were acquired pre- and post-injection of the intravascular iron oxide compound Feraheme (4 mg/kg; ferumoxytol, AMAG Pharmaceuticals, Inc. Waltham, MA, USA) [27]. Information from dynamic scans were processed by BALDERO (blood agent level dependent and extravasation relaxation overview) model to derive measurements of tissue permeability to proteins/albumin (K^trans^) and the proportion of contrast reagent present in extravascular/extracellular space (v_e_).

### Cell culture

Granulosa cells (LGCs) were collected at the time of follicle aspiration and pooled for culture by female: LGCs from random females who did not receive Avastin before aspiration (LGCs; *n* = 7), and from all females who received Avastin before hCG (LGCs + Avastin; *n* = 6). Red blood cell contaminants were removed from aspirates using Percoll gradient (30%) centrifugation as previously described [28]. The viability of LGCs was assessed before plating by Trypan blue exclusion and cell numbers determined by hemocytometer. LGCs (19.5 ± 4 × 10^6^ viable LGCs/female) were dived equally into 6 well plates (1 plate/female) previously coated with charcoal-treated fetal calf serum (Sigma-Aldrich, St. Louis, MO, USA) and cultured for 24 h in defined media similar to previous studies [28], containing FSH (2.5 ng/ml; EMD Serono, Rockland, MA, USA) and LH (100 ng/ml; A. Parlow, National Hormone and Peptide Program, UCLA Medical Center, USA). Media were collected, pooled by female, and stored at −80 °C until assayed for hormones as described below.

### Hormone measurements

E2 and P4 were analyzed by automated clinical platform Roche Cobas e411 at the ONPRC Endocrine Technology Support Core Laboratory. Levels of VEGFA and angiopoietins 1 and 2 (ANGPT1 and ANGPT2) assayed with Human Quantikine Elisa kits (R&D Systems, Inc., Minneapolis, MN, USA) [29].

### Statistics

Mixed Models function of SAS with repeated measures was used to interrogate changes in serum P4 levels, ovarian and uterine (whole uterus, junctional zone, and endometrial) blood volume (BV) and vascular flow (VF) by time from hCG or days SEP (version 9.4, SAS Institute Inc. Cary, NC, USA). Differences between individual time points and treatments were tested by Least Squared Means function of SAS. Discrete variables (ovarian permeability (K^trans^ and v_e_), oocyte totals/stages, E2 at time of hCG, intrafollicular and media VEGFA, P4, ANGPT1, ANGPT2) were analyzed by One-way ANOVA function of SAS, differences between individual treatments were analyzed by Fisher’s Least Squared Difference (LSD) test. To correct for heterogeneity of variances, K^trans^ and v_e_ data were first coded by 1000 and then Log_10_ transformed before analyses.

## Results

### Treatment with Avastin pre-hCG/post-hCG

**Fig. 1:**
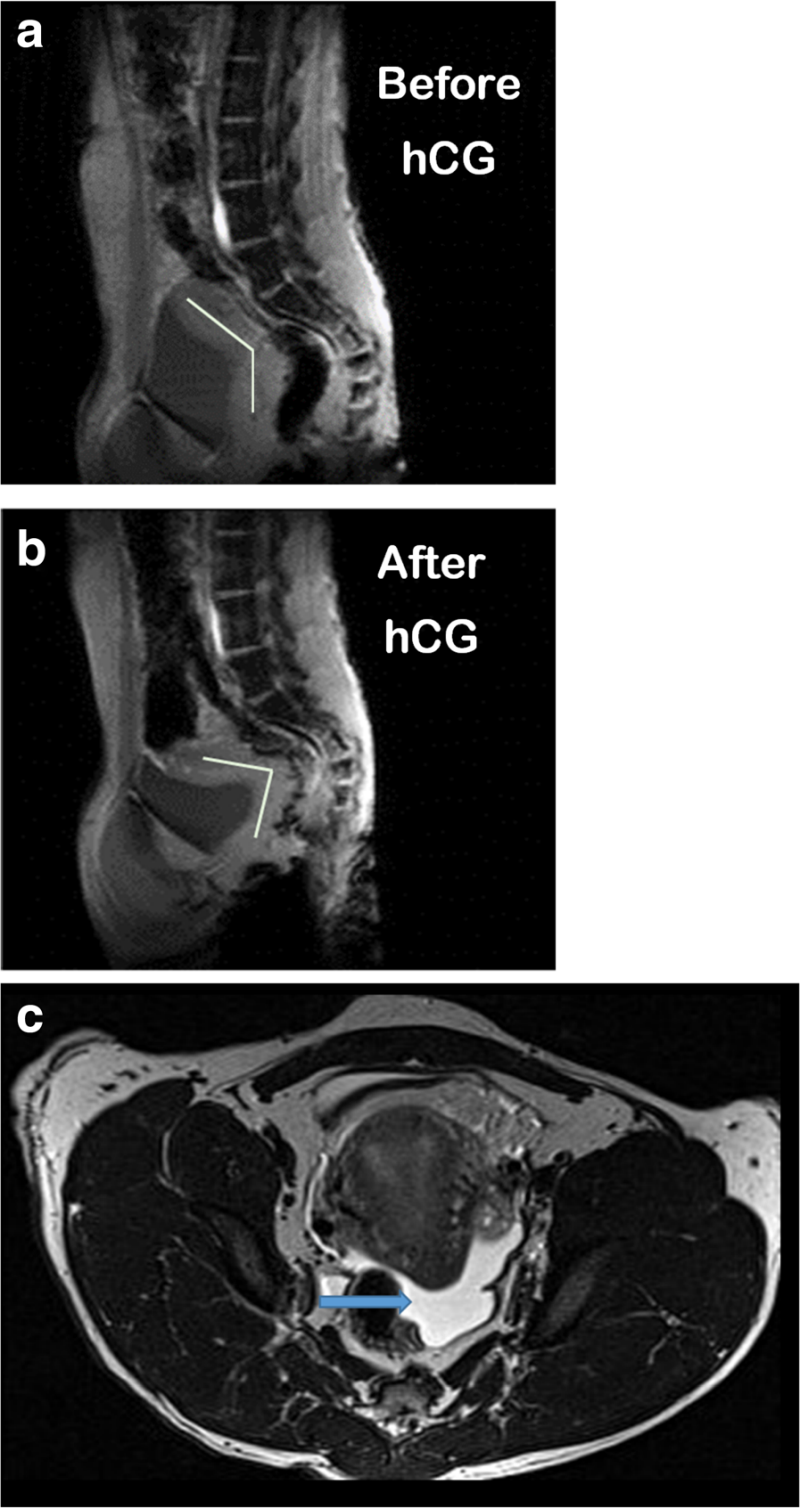
Rhesus macaques show symptoms of moderate OHSS following hCG administration in COS cycles. Magnetic resonance imaging (MRI) depicting pelvic anatomy before (**a**) and after (**b**) hCG administration in COS cycles in one representative macaque female (tissue density is depicted in *grey scale*). The reproductive tract (uterus and vaginal canal) is highlighted by *light grey line*. Presence of ascites (fluid is *bright white*) is depicted in this female after exposure to hCG (**c**) and indicated by *blue arrow*

**Fig. 2:**
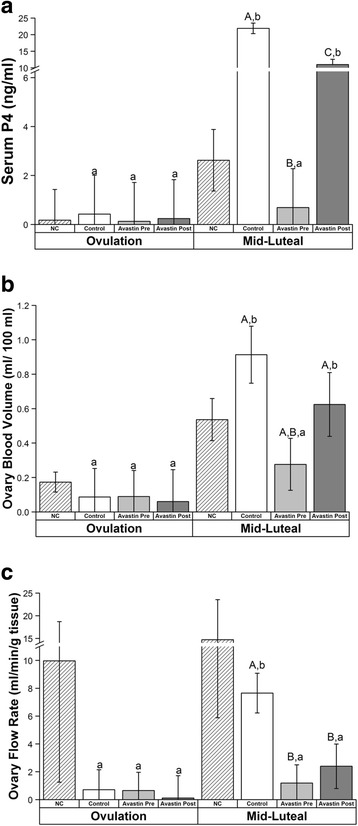
Effects of VEGFA neutralization during COS cycles; ovarian vascular flow/volume. Serum progesterone (P4; **a**), ovarian blood volume (BV; **b**), and ovarian vascular rate of flow (VF; **c**) in control COS cycles and COS cycles + anti-VEGFA (Avastin) treatment pre-hCG (Avastin Pre) and post hCG (Avastin Post). Values observed during natural menstrual cycles in these same females (NC) are depicted as a reference. For details of treatments see Materials and Methods. Upper case letters indicate differences (*P* < 0.05) between COS treatment groups at mid-luteal phase and lower case letters indicate differences (*P* < 0.05) within each treatment group between ovulation and mid-luteal phase

Stimulation characteristics of COS animals are presented in Table 1. There were no differences in peak E2 levels, numbers of oocytes or stages of oocytes (metaphase II/MII, metaphase I/MI, germinal vesicle/GV) recovered (all *P* > 0.2) between Control, Avastin Pre-hCG and Avastin Post-hCG COS cycles. However, the presence of ascites fluid was noted by MRI analyses performed 6–8 days post-hCG (“mid-luteal” phase) in only 33% of females exposed to Avastin pre-hCG, compared to 80% of Control (Example representative control female with abdominal ascites, Fig. 1) and 75% of Avastin Post-hCG females. Serum P4 concentrations significantly increased in Control COS cycles by mid-luteal phase compared to Pre-hCG trigger (Fig. 2a). However, this increase was abrogated by Avastin treatment Pre-hCG. Serum concentrations of P4 also increased above baseline in females exposed to Avastin Post-hCG, however peak levels of P4 were significantly lower than Control COS cycles. The luteal phase in natural menstrual cycles was 18 ± 0.6 days (day of mid-cycle E2 peak = day 0, until onset of menses), while the luteal phase of control COS females (from day of hCG until onset of menses or P4 concentrations below 1 ng/ml for 3 days) was only 12.6 ± 1.3 days and Avastin post-hCG was 11.7 ± 2.6 days. Females treated with Avastin pre-hCG had a luteal phase of 6.9 ± 2.4 days in duration. Natural menstrual cycle data are included for comparison with COS, however, during DCE-MRI protocols bolus injection of contrast reagent failed in 6 of 8 animals due to the presence of a back-flow regulator on the IV-setup. The backflow regulator was removed from the setup in subsequent scanning sessions. Both CEUS and DCE-MRI data from natural cycles were not included in final statistical analyses.

**Table 1:** Stimulation Characteristics of Rhesus Females (mean ± SEM or %)

	Control	Avastin Pre-hCG	Avastin Post-hCG
Peak E2 (pg/ml)^1^	3813 ± 631.7	5063 ± 727	4911.6 ± 827
Total # Oocytes^1^	37 ± 3.5	73.4 ± 10.2	73 ± 24.9
MII^1^	16.3 ± 1	29.5 ± 5.9	36 ± 11
MI^1^	12 ± 1.5	19.7 ± 2.2	14.2 ± 4.1
GV^1^	7.25 ± 2.1	19 ± 2.9	12 ± 4.9
Ascites Fluid (% Total)	4/5 (80%)	2/6 (33%)	3/4 (75%)

^1^ *P* > 0.2

**Fig. 3:**
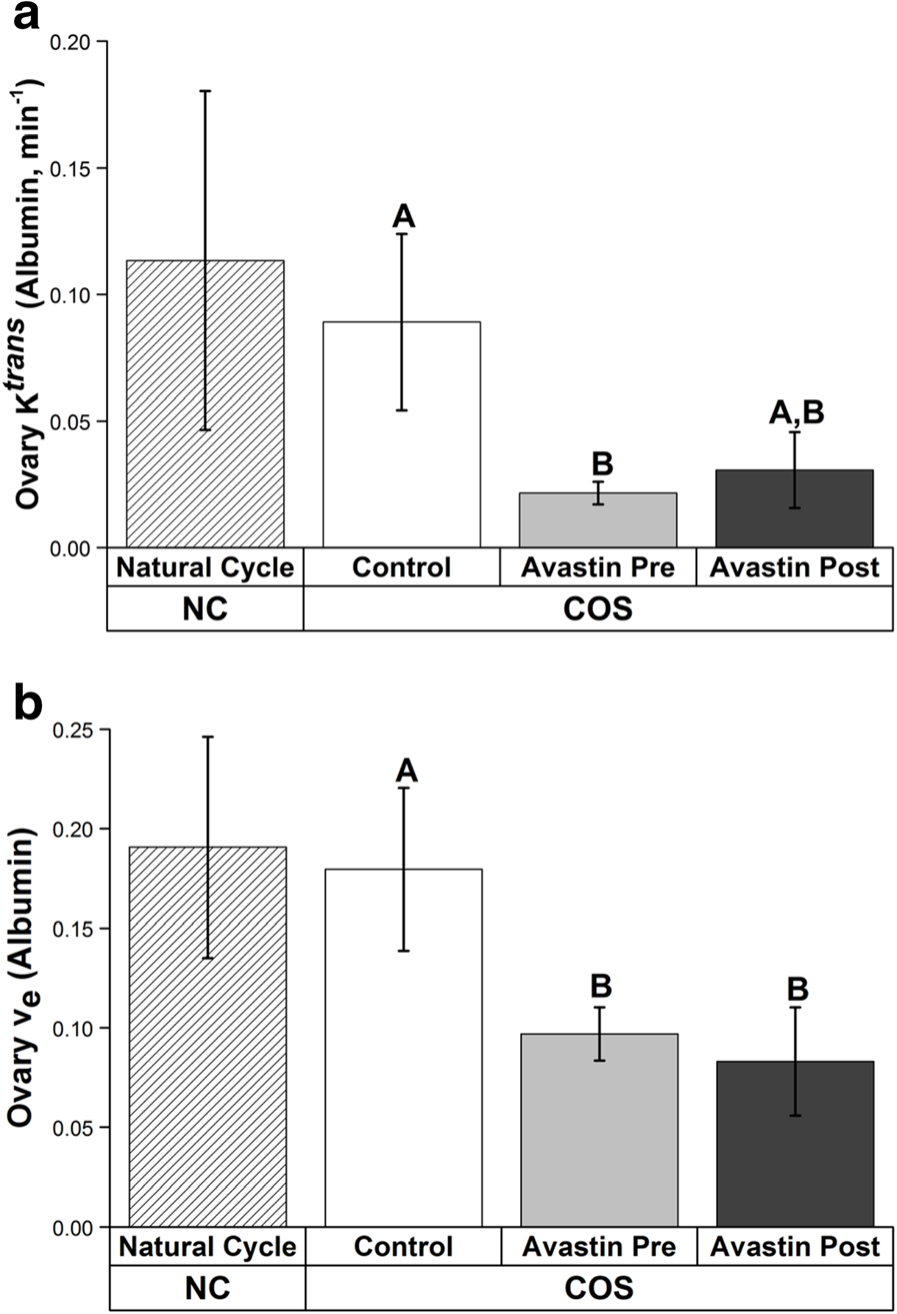
Effects of VEGFA neutralization during COS cycles; ovarian permeability. DCE-MRI analyses at mid-luteal/luteal phase of albumin flux through ovarian tissue in COS control cycles and COS cycles with and without Avastin treatment pre-hCG (Avastin Pre) and post-hCG (Avastin Post). Data from the CL of natural menstrual cycle (NC) are included as reference. **a** Estimation of ovarian permeability to albumin (K^trans^) and **b** estimate of gadolidium-conjugated albumin present in extravascular/extracellular space (v_e_). Different uppercase letters indicate differences (*P* < 0.05) between COS Control, Avastin Pre and Avastin Post treatment groups

Ovarian blood volume (BV) increased in Control and Avastin Post-hCG and this increase was prevented by Avastin Pre-hCG (Fig. 2b, Additional file 1: Figure S1A). Ovarian vascular flow (VF) was significantly reduced at mid-luteal phase by Avastin treatment both Pre- and Post-hCG trigger; only Control cycles displayed increased vascular flow at mid-luteal phase (Fig. 2c, Additional file 1: Figure S1A). There were no effects on uterine vascular function (BV or VF) as measured in the entire uterus, the basalis zone (junction) or the endometrium in COS cycles (all *P* > 0.2). The rapid rate of uterine vascular flow combined with a large uterine blood volume rendered estimation of flux of serum albumin by DCE-MRI modeling unreliable, since all measurements resembled those obtained for arteries and veins. However, successful measurements were obtained from the small capillary beds in ovaries of COS cycles. Ovarian permeability (estimated by K^trans^) was reduced by pre-treatment with Avastin Pre-hCG compared to Control COS cycles (Fig. 3a, Additional file 1: Figure S1B). Estimation of the proportion of serum albumin present in extravascular space within the ovary (v_e_) was reduced by Avastin treatment both Pre- and Post-hCG bolus.

### Follicular fluid and in vitro (LGC) analyses of Avastin treatment

**Fig. 4:**
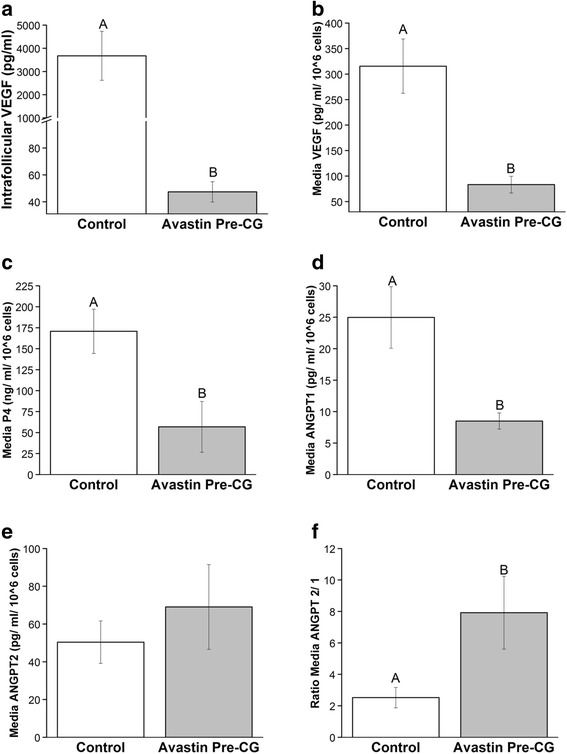
Local effects on ovarian angiogenic factors by VEGFA neutralization in COS cycles. Intrafollicular VEGFA (**a**), and LGC production of VEGFA (**b**), progesterone (P4; **c**), ANGPT1 (**d**), ANGPT2 (**e**), and ratio of ANGPT2:1 (**f**) as detected in media after 24 h of culture in aspirates from control COS and anti-VEGFA (Avastin) treated females. See Methods for further details. Different uppercase letters indicate significant (*P* < 0.05) differences between Control and Avastin Pre-CG treatment groups

The stimulation characteristics of females exposed to Avastin Post-hCG were statistically similar to COS females (Table 1; aspiration performed before Avastin treatment). Therefore, follicular fluid from two randomly chosen Avastin Post-hCG females were included in these analyses (Additional file 2: Table S1). No differences in oocyte parameters were detected in these females, or in intrafollicular levels of P4, ANGPT1, ANGPT2 or ratio of ANGPT2/1 (Additional file 2: Table S1). However, intrafollicular levels of VEGF were significantly reduced 78-fold by the single IV bolus of Avastin (Fig. 4a).

Avastin treatment in vivo reduced VEGF production by cultures of LGCs 4-fold (Fig. 4b), and production of P4 was also reduced compared to Control LGCs (Fig. 4c). The decline in P4 production by Avastin-exposed LGCs mimics the reduced P4 produced in Avastin Pre-hCG females observed at mid-luteal phase in vivo (Fig. 2a). Angiogenic potential of these LGCs was determined by measurement of ANGPT1 and ANGPT2. There was significantly less ANGPT1 is produced by Avastin-exposed LGCs (Fig. 4d), and while production of ANGPT2 is not impacted by Avastin treatment in vivo (Fig. 4e), the ratio of ANGPT2/ANGPT1 is elevated in these LGCs compared to unexposed Controls (Fig. 4f).

### Treatment with Avastin in COS cycles +SEP

**Fig. 5:**
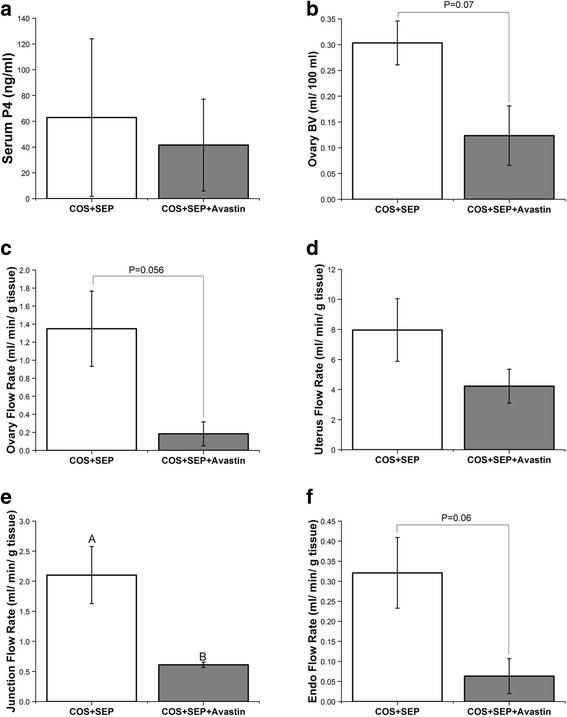
Effects of VEGFA neutralization during COS + SEP cycles. Serum progesterone (P4; **a**), ovarian blood volume (BV; **b**), and ovarian vascular rate of flow (VF; **c**), uterus VF (**d**), junction (basalis) VF (**e**), and endometrial VF (**f**) in control COS + SEP cycles and COS + SEP cycles + anti-VEGFA (Avastin) treatment. For details of treatments see Methods. Upper case letters indicate differences (*P* < 0.05) between COS + SEP and COS + SEP + Avastin treatment groups

Animals were analyzed near time of peak P4 response to reduce animal to animal variation. Peak P4 response to SEP was not different between SEP and SEP + Avastin treatments (Fig. 5a). Ovarian BV tended to be reduced by Avastin exposure during SEP (Fig. 5b), and a strong trend towards reduced ovarian VF was found (Fig. 5c). Unlike COS only cycles, VF in the basalis zone/junction was significantly reduced (Fig. 5e) and endometrial VF tended to be reduced (Fig. 5f) in SEP + Avastin treatment compared to SEP cycles. There were no differences in overall uterine VF (Fig. 5d) or BV in any uterine compartment (all *P* > 0.2; data not shown). There were no differences in ovarian permeability to serum albumin by DCE-MRI analyses between SEP and SEP + Avastin cycles (K^trans^ *P* > 0.15 and v_e_ *P* > 0.4; data not shown).

## Discussion

The goals of this study were three-fold, 1) verify that VEGFA is the main factor contributing to increased ovarian hyperpermeability following hCG-exposure in COS cycles, 2) determine if intravenous administration of a humanized monoclonal antibody targeting VEGFA (Avastin) during COS cycles provided a viable model to interrogate ovarian angiogenesis, and 3) to determine if hCG-induced VEGFA contributes to ovarian hyperpermeability/OHSS symptoms during embryo implantation/early pregnancy. Since these studies are of interest as potential targets for OHSS therapies, the effects of Avastin treatment following COS on uterine blood flow were also analyzed following this treatment regimen. The actions of VEGFA to induce abdominal ascites, the primary symptom of OHSS, appear to manifest before or near the time of aspiration. There is a possibility the shorter period between administration and evaluation was not long enough to halt any processes leading to ascites development in the Avastin Post-hCG group. However, a more likely scenario is that treatment pre-hCG neutralized the initial rise in VEGFA observed in the ovulating antral follicle [6, 30]. These data are similar to previous reports of minimal effects following intra-luteal injection of VEGF antagonists into the primate CL of the natural menstrual cycle of subsequent luteal function [6]. Although a second rise of VEGFA is observed between the early and mid-late luteal phase [31], this latter rise may only support sustained function of the primate luteal capillary bed.

These studies also demonstrate that intravenous neutralization of VEGFA before ovulatory stimuli in COS cycles effectively reduces the onset of OHSS-like symptoms in rhesus macaques. Blockage of VEGFA action was reported in rhesus monkeys by direct injection of VEGF antagonists into ovulating follicles was found to prevent luteal capillary bed formation [32]. These studies now demonstrate that a single intravenous injection of Avastin is also highly effective in preventing the rise of intrafollicular free VEGFA following an ovulatory stimulus in COS cycles. Use of intravenous administration of Avastin, combined with minimally invasive ultrasound and MR-imaging of ovarian vascular parameters provide a desirable experimental model for primate studies. The reduced ovarian blood flow, volume, and vascular permeability reported in this study is likely a manifestation of disrupted vascularization processes in females treated pre-hCG. Avastin treatment in the early luteal phase also resulted in lower ovarian vascular flow and reduced P4 production by luteal tissue. Levels of VEGFA mRNA are elevated in macaque CL at mid-luteal phase [4]; our data suggest that VEGFA acts to maintain the high rate of blood flow and permeability within primate luteal tissue to facilitate efficient secretion of luteal steroids. Women administered a non-specific VEGFA/dopamine antagonist cabergoline on the day of hCG trigger also report reduced risk for development of early onset OHSS [33], and cabergoline reduced leakage of a small molecular weight contrast reagent (Gd-DTPA-BMA) out of ovaries when administered starting the day of hCG [34]. We now show that leakage of the large molecular weight protein albumin induced by hCG exposure in rhesus monkeys undergoing COS is reduced by direct neutralization of VEGFA both before and immediately after hCG exposure. Since the animals in this study only developed moderate OHSS symptoms of abdominal ascites, it remains to be determined if progression from moderate to severe OHSS could be prevented by Avastin treatment post-hCG in women. Given this limitation, however, this less-invasive primate model can be used in future investigations to evaluate other ovarian vascular processes activated by VEGFA contributing to ovarian hyperpermeability and development of abdominal ascites.

Dramatically reduced VEGFA levels were observed in follicular fluid following Avastin treatment prior to hCG bolus in COS cycles. In other systems, VEGFA can be sequestered in cells and released by the breakdown of the cellular matrix by matrix metalloproteinases (MMPs) [35]. It is important to note the VEGFA assay used to analyze intrafollicular levels of VEGFA detects only free VEGFA [36], so the absolute level of VEGFA (free and matrix bound) still may be elevated in these ovaries. But, reduction in free VEGFA levels was also detected in media from granulosa cell (LGC) cultures exposed to VEGFA mAb in vivo. This reduction may also be the result of diminished release of membrane-sequestered VEGFA by these LGCs. Regardless, the LGC culture data suggest VEGFA actively regulates follicular ANGPT1 production. The levels of ANGPT2 rise in primate follicles at the time of antrum formation [37]. Since intrafollicular levels of ANGPT2 are higher than ANGPT1 in ovulatory follicles [38], the ANGPT2:1 ratio in Controls is consistent with the literature. In primate granulosa cells production of both ANGPT1 and ANGPT2 is insensitive to oxygen tension [37]. Therefore, regulation of ANGPT1 by VEGFA is not necessarily a function of in vivo Avastin-treated LGCs originating from a follicle at a more hypoxic-state than control LGCs. This elevated ANGPT2:1 ratio observed in LGC cultures from the Avastin Pre-hCG group provides an insight into the potential mechanism of action by which vascular flow, volume, and permeability are reduced by Avastin. The actions of ANGPT1 in endothelial cells are mediated via the Tie 2 receptor tyrosine kinase; ANGPT2 acts as a competitive antagonist to prevent ANGPT1 from binding to the Tie2 receptor [39]. ANGPT2 functions to destabilize endothelial cell junctions: when local ANGPT2 levels are higher than ANGPT1 and VEGFA is present this results in neovascularization, but if VEGFA is absent, this ratio leads to endothelial cell apoptosis [39, 40]. Levels of ANGPT2 are already higher than ANGPT1 during ovulation, and when combined with high VEGFA levels these induce luteal angiogenesis [6, 41]. In Avastin pre-hCG treated females high levels of ANGPT2 in the absence of elevated VEGFA in follicles likely prevents normal formation of the luteal vascular bed, via induction of apoptosis in endothelial cells and vessel degeneration. When VEGFA is neutralized at later time point (i.e. post-hCG and SEP), the local ANGPT2:1 ratio is again disrupted and degeneration of the luteal vascular bed is induced, albeit to a lesser extent given the reduced number of proliferating endothelial cells at these later stages [42].

Simulated early pregnancy (SEP) studies were performed to determine if CG-driven VEGFA is also a major component of late onset OHSS. Previous studies in women have suggested that CG stimulates further angiogenic processes in the mid-late luteal phase [43]. However other studies in nonhuman primates report contradictory results [42]. What is consistent between studies in women and nonhuman primates is high levels of VEGFA mRNA are maintained in luteal tissue by CG administration compared to regressing CL [23, 43]. Ovarian vascular flow rate was lower in SEP cycles compared to COS cycles (Control group). And, while the average reduction in blood volume and vascular flow by Avastin treatment during SEP cycles was greater than in the Avastin Post-hCG group, the overall blood volume present within the ovary during SEP cycles was lower than control cycles alone. Moreover, unlike control cycles, Avastin treatment did not lower ovarian permeability to albumin during SEP. These data suggest that VEGFA appears to enhance overall blood flow and volume throughout luteal tissue of early pregnancy, however it does not sustain luteal vascular function in early pregnancy. This finding is consistent with reported differences of endothelial cell proliferation makers within primate CL during the luteal phase and SEP [42]. Other angiogenic factors induced by CG may be more critical to the development of late onset OHSS in women. Serum P4 levels were not affected by Avastin treatment during SEP, the variation in response to SEP by females indicates increased treatment group variability. Combined with the lower numbers of females undergoing SEP, it is clear larger studies would be needed to determine any subtle effects on the ovarian vasculature of VEGFA neutralization in SEP.

An important consideration for extending these findings to use in women at risk for OHSS seeking to become pregnant is to determine any effects on uterine function. No detrimental effects of Avastin treatment in COS cycles 3–4 days post-hCG were detected on uterine vascular function. Serum levels of P4 rose high enough in females treated with VEGFA mAb Avastin post-hCG that vascular function of the secretory-stage endometrium was not impacted compared to control COS cycles (as measured in the entire uterus, the basalis/junctional zone between the endometrium and myometrium, and within the endometrium). It is important to note that the luteal phase in women following COS is often of shorter duration than that of natural cycles [44], and we also report shorter luteal phases in all of the COS-treated rhesus monkeys. Analyses of uterine vascular function were performed on days 6–8 post-hCG in COS cycles, and at this time 4/5 control and 3/4 treated females had P4 levels >1 ng/ml; 3 days after levels fall below 1–0.5 ng/ml menses is typically detected in rhesus monkeys [45]. These data indicate that uterine vascular function was comparable between control and Avastin post-hCG cycles, and this was not due to early initiation of menses/endometrial shedding in control COS females on the day of analysis. It is unknown if the morphology of the uterine spiral arteries in this treatment group is comparable to that of natural menstrual cycles during the secretory phase with expected endometrial gland expression of progesterone and estrogen receptors indicating the endometrium is receptive to an implanting embryo [46, 47].

But, detrimental effects of Avastin in the junction/basalis zone at the base of the spiral arteries was observed when hCG was used to mimic the presence of an implanting embryo. Expression of VEGF protein is reported in blood vessels of the endometrium in women only during early pregnancy [48], although VEGF is reportedly present in epithelial and stromal cells of both proliferative and secretory endometrium [48, 49]. The data reported here are consistent with these sites of action for VEGFA in the uterus: there was compromised vascular function only of those spiral arteries exposed to Avastin in the presence of SEP. These data obtained during SEP following COS cycles are also consistent with reports of implantation defects in rhesus females treated with VEGFA mAbs in natural conception cycles during the window of implantation [50, 51], and show that this treatment regimen would likely not be appropriate for women who wish to become pregnant in fresh cycles. However, future studies could compare uterine vascular function in women in women undergoing embryo transfer after cryopreservation treated with either VEGFA mAbs or cabergoline. These data also serve to emphasize that evaluation of anti-angiogenic agents for OHSS therapies should be evaluated in context of both pregnant and non-pregnant primates.

## Conclusions

Intravenous direct neutralization of VEGFA before hCG exposure in COS cycles prevents the development of OHSS symptoms in rhesus monkeys. Avastin treatment in vivo alters the angiogenic potential of LGCs retrieved following COS. The elevated ANGPT 2/1 ratio observed in vitro in the absence of elevated VEGFA both in vitro and in vivo indicates the angiogenic signaling environment is altered to promote vessel degeneration following Avastin exposure. While there were minimal effects of Avastin treatment on uterine vascular function in COS cycles, Avastin treatment altered uterine vascular function in this model of SEP. Further studies utilizing this nonhuman primate model will provide insights into VEGFA-induced factors regulating aberrant ovarian angiogenesis contributing to development of ovarian hyperstimulation syndrome during COS cycles in women.

## Additional files

Additional file 1: Figure S1. Parametric maps of ovarian CEUS and DCE-MRI analyses. **Figure S1 A**: False-color images of ovarian blood volume (BV) and vascular rate of flow (VF) as determined by CEUS analyses for each treatment group (see materials and methods for details). Black areas within ovarian flow shows areas of low vascularization. **Figure S1 B**: False-color images of ovarian vascular permeability to albumin (K^trans^) and proportion of contrast reagent present in extravascular/extracellular spaces (v_e_) as determined by DCE-MRI analyses of COS only cycles (see materials and methods for details). Note the differences in maximal values for scale between groups. (TIFF 3928 kb)

Additional file 2: Table S1. Follicular Fluid/Aspiration Analyses of Control COS and COS + Avastin Pre-hCG Stimulations. Excel Spreadsheet. (XLSX 11 kb)
