# Supplementary figures and images for: Intravenous neutralization of vascular endothelial growth factor reduces vascular function/permeability of the ovary and prevents development of OHSS-like symptoms in rhesus monkeys
Source: J Ovarian Res. 2017 Jul 6;10:41. doi: 10.1186/s13048-017-0340-5 (PMC5501270; doi:10.1186/s13048-017-0340-5)

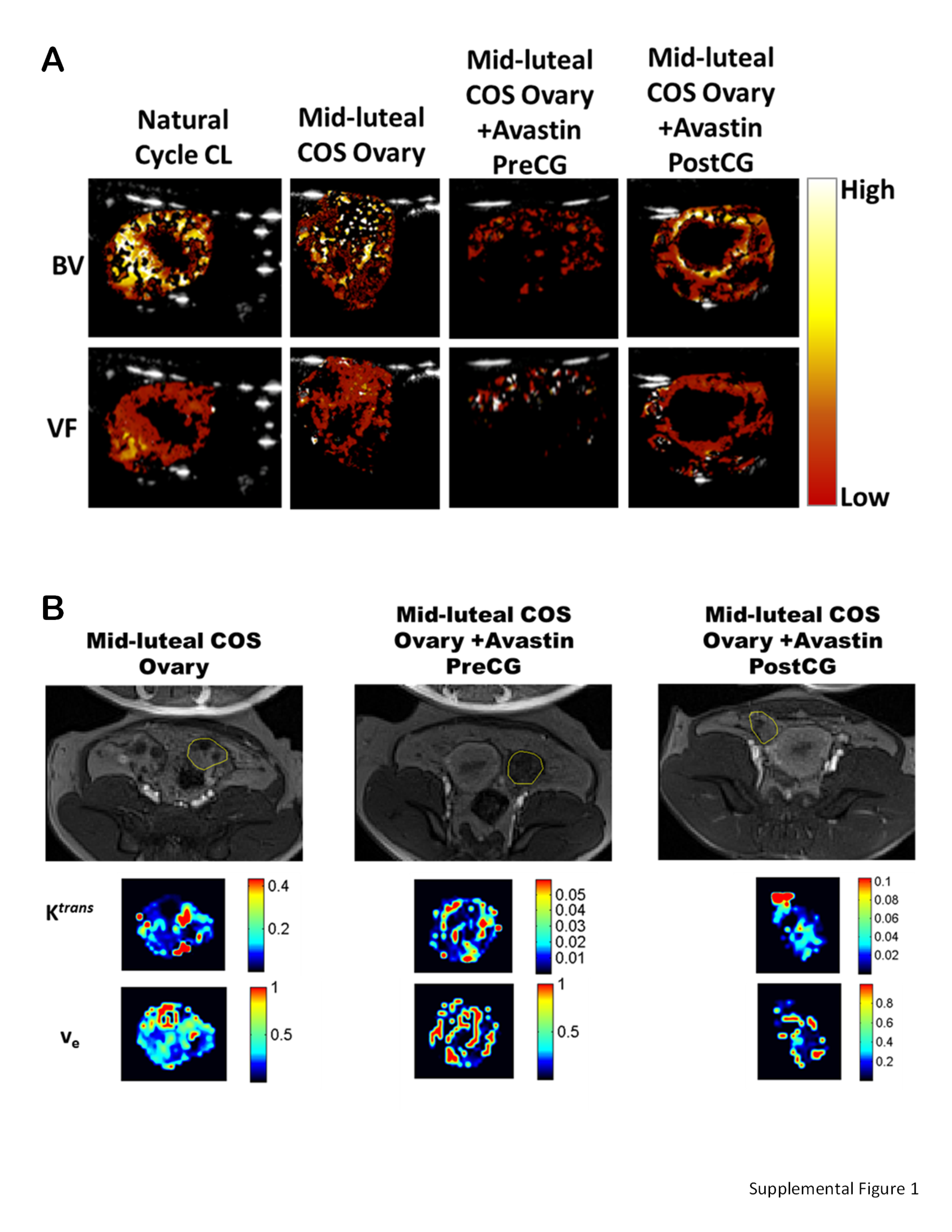

Supplement: Supplementary file 1 — Parametric maps of ovarian CEUS and DCE-MRI analyses. Figure S1 A: False-color images of ovarian blood volume (BV) and vascular rate of flow (VF) as determined by CEUS analyses for each treatment group (see materials and methods for details). Black areas within ovarian flow shows areas of low vascularization. Figure S1 B: False-color images of ovarian vascular permeability to albumin (Ktrans) and proportion of contrast reagent present in extravascular/extracellular spaces (ve) as determined by DCE-MRI analyses of COS only cycles (see materials and methods for details). Note the differences in maximal values for scale between groups. (TIFF 3928 kb) [file 13048_2017_340_MOESM1_ESM.tif]
